# Fretting properties of biodegradable Mg-Nd-Zn-Zr alloy in air and in Hank’s solution

**DOI:** 10.1038/srep35803

**Published:** 2016-11-04

**Authors:** Wenting Li, Nan Li, Yufeng Zheng, Guangyin Yuan

**Affiliations:** 1 Department of Materials Science and Engineering, College of Engineering, Peking University, Beijing 100871, China; 2 National Engineering Research Center of Light Alloys Net Forming, Shanghai Jiao Tong University, Shanghai 200240, China

## Abstract

Fretting is a significant cause for the failure of orthopedic implants. Currently, since magnesium and its alloys have been developed as promising biodegradable implant materials, the fretting behavior of the Mg alloys is of great research significance. In this study, a Mg-Nd-Zn-Zr alloy (hereafter, denoted as JDBM alloy) was selected as experimental material, and its fretting behaviors were evaluated under 5 N, 10 N and 20 N normal loads with a displacement of 200 μm under the frequency of 10 Hz at 37 °C in air and in Hank’s solution, respectively. The results indicated that while the friction coefficient decreased with the increment of the normal load, the wear volume of the alloy increased with the increment of the normal load both in air and in Hank’s solution. Both the friction coefficients and the wear volume of the fretting in Hank’s solution were much lower than those in air environment. The evolution trend of friction coefficients with time had different performance in air environment and the Hank’s solution group. Although oxidation occurred during the fretting tests in Hank’s solution, the damage of JDBM alloy was still reduced due to the lubrication effects of Hank’s solution. Moreover, the addition of Fetal bovine serum (FBS) could act as lubrication and result in the reduction of the fretting damage.

Fretting is one of the most deleterious mechanisms for the degradation of materials. Fretting occurs when two surfaces are in contact with each other in “small” amplitude displacements (on the order of microns), that are with a total amplitude smaller than the contact width, resulting in damage to the surfaces in the contact region^1^. This fretting damage is derived from two main mechanisms: fretting wear and fretting fatigue^2^. Fretting wear results in the generation of debris and mismatching between contacting surfaces. Rapid crack nucleation and propagation resulted from fretting fatigue can lead to catastrophic failure of the components^3^. Besides, in some cases, environmental factors are of importance in the fretting resulting in corrosive and mechanical damages, which is known as fretting-corrosion^4^. Understanding fretting behavior is a prerequisite for using new materials that will be used in a contact situation with vibration.

Fretting frequently happens in machines and engineering structures^4^. It also appears in orthopaedic implants when patients take physical activities^5^, always being identified at the stem/neck and neck/head contacts of modular implants, the stem/bone and stem cement interfaces of cemented and uncemented implants, and the screw/plate junction of fixation plates^6^. Fretting occurring in these sites results in aseptic loosening, early loss of mechanical integrity and eventual failure of the implant, causing re-surgery and suffering to the patients^7^,^8^. Fretting also accelerates the corrosion of implants that exposed to the physiological fluid due to the mechanical disruption of the passive surface oxide layer^9^. The release of corrosion products such as heavy-metal ions and particulate debris into the surrounding tissue could cause adverse biological reactions^10^. Early in 1913, Hey-Groves clearly observed that when a plate was attached to a bone to fix the fractured bone fragments, due to the movement of patients, there inevitably existed slight but constantly movement that would cause degradation of mechanical property of the implant^11^. Then in 1962, Cohen investigated the reason for corrosion occurring at screw-plate interfaces and concluded that fretting could contribute to the corrosion^12^. In 1969, Colangelo and Greene claimed that fretting corrosion was seen on more than 90% of plates and screw implants made of 316 stainless steel^9^. Waterhouse observed the failures of metallic components in human body resulted from fretting fatigue between the underside of the screw head and the countersink of the hole^13^. Some researches were carried out, focusing on the fretting behaviors of CP titanium and titanium alloys which were widely used as bone implants^14^,^15^,^16^,^17^,^18^,^19^. For example, Animesh Choubey *et al*. reported abrasion and cracking were the predominant wear mechanisms of CP titanium and titanium alloys against bearing steel under the condition of 10 N normal load, frequency at 10 Hz with the displacement of 80 μm in Hank’s solution^14^. N. Diomidis *et al*.^20^ studied the fretting-corrosion behaviors of four different titanium alloys in Hank’s solution and concluded that the effect of the mechanical properties of the alloys was not important on the fretting response and wear volumes. The author also evaluated the effect of synovial on fretting behavior and deduced that the presence of synovial constituents lubricated the contact making slip easier.

However, the most extensively used artificial implant metallic biomaterials orthopedic such as stainless steel, titanium and CoCrMo alloys in orthopedic fields are associated with stress shielding phenomena owing to their high elastic modulus compared to human bone^21^, and require a second intervention for implant removal particularly for paediatric and adolescent patients. Magnesium alloys have been widely studied recently for their potential applications as biocompatible, osteoconductive, biodegradable implant materials within bone^22^,^23^,^24^,^25^,^26^,^27^,^28^. Besides, stress shielding phenomena is greatly alleviated due to the close of elastic modulus of magnesium alloys to that of bone. Magnesium alloys play an active role in the bone response process^29^. The results obtained from current *in vitro* and *in vivo* tests indicated that JDBM alloy satisfied the general requirements for biodegradable biomaterials^30^,^31^,^32^. JDBM alloy exhibits enough strength, excellent elongation and corrosion resistance after hot extrusion and heat treatment^30^,^33^. Furthermore, JDBM alloy effectively enhances the corrosion resistance, biocompatibility and antimicrobial properties of Mg by alloying with the proper amount of Zn, Zr and Nd both *in vitro* and *in vivo*^30^. Understanding the fretting behaviors of JDBM is a key to using this prospective biodegradable material in orthopedic application. Previous studies had shown that the fretting behavior of AZ91D and AM60 magnesium alloys in dry condition was influenced by normal load and slip amplitude^2^,^34^, and fatigue strength of AZ61 alloy reduced because of fretting^35^. Yet as a promising biodegradable metal, the fretting behavior of magnesium alloys in a physiological environment has not been reported till now. In the present study, the fretting behaviors of JDBM in air and in Hank’s solution were comparatively investigated.

Firstly, fretting tests were performed in air as a reference environment. Then the fretting tests were performed in Hank’s solution supplied with or without fetal bovine serum (FBS) that simulated the physiological liquid in order to investigate the effect of Hank’s solution and proteins on fretting behaviors of JDBM alloy. Dissipated energy and wear damages were used to evaluate the effect of the aqueous environment on damage of JDBM alloy surface during fretting. Ceramics spheres (Si_3_N_4_) and JDBM alloy plates were used in this study to form ball-on-flat geometry. In fact, the interface of counterpart has a big influence on the fretting behavior of JDBM alloy. Silicon nitride ceramic was selected because it has shown promising properties as wear resistant material^36^. Its damage in this study was far less severe than that of JDBM alloy, so it was easy to focus on the different environmental effects on fretting behavior of JDBM alloy. Considering the potential application of JDBM alloy in bone implant field, normal load lie within the range from 5 N to 20 N, which is approximately 100 MPa by Hertz contact, so as to obtain approximately the same range of contact pressure between the screw head and the plate hole^37^. In addition, the pressures can also occur between the plate and bone^38^.

## Results

### Friction coefficients

**Figure 1:**
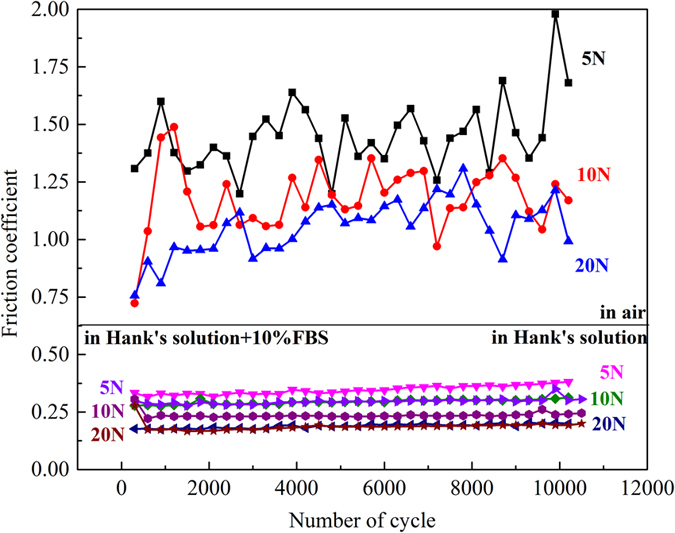
Evolution of friction coefficients of JDBM alloy with the normal load of 5 N, 10 N and 20 N in air and in Hank’s solution with and without FBS.

The evolution of friction coefficients with cycles in air and in Hank’s solution under 5 N, 10 N and 20 N normal load are shown in Fig. 1. Both in air and in Hank’s solution, the friction coefficients, more exactly the ratio of tangential force to normal force, basically decreased with the increase of normal load. This phenomenon was also observed in the reference^2^. Figure 1 illustrates that the Hank’s solution reduced friction coefficient of JDBM alloy by a factor of 4 in comparison to that in air, indicating that Hank’s solution acted as a lubricant. Besides, when FBS was added into Hank’s solution, the friction coefficient became lower than that in Hank’s solution without addition of FBS under 5 N and 10 N normal load. However, when the normal load reached 20 N, no evident difference of the friction coefficient in simulated body fluid with and without FBS was observed. The evolution of friction coefficients in air can be divided into two stages: initial stage and stable stage. The initial stage resulting from the damage of the smooth surface can be further divided into ascending stage and regressive stage, especially when the normal load was 5 N or 10 N. When the normal load reached to 20 N, the contact surface was destroyed so quickly that only one stage can be observed. The friction coefficient during the stable stage fluctuated around a constant. The evolution of friction coefficients in Hank’s solution was quite different with only one stage presented. The friction coefficients faintly increased with increase of the cycle numbers and were more stable compared to that in air. The addition of FBS did not change the evolution of friction coefficient.

### Fretting logs

**Figure 2:**
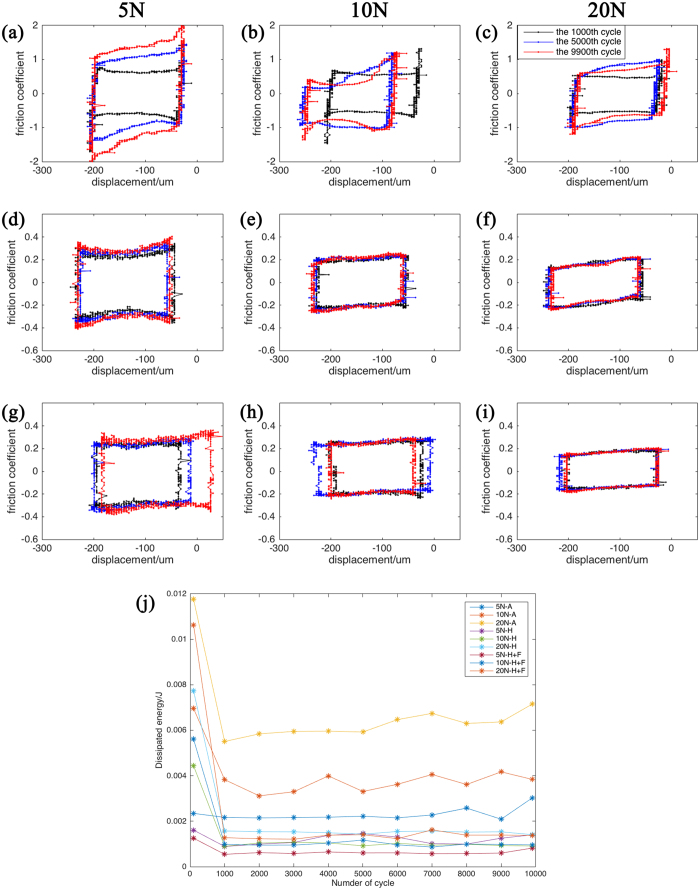
Fretting log diagrams of JDBM with the normal load of 5 N, 10 N and 20 N in air (**a**–**c**) and in Hank’s solution (**d**–**f**) without addition of FBS and with FBS (**g**–**i**) when the number of cycles are 100, 5000 and 9900 respectively. And the evolution of dissipated energy with time during the fretting tests (**j**).

During the fretting tests, variations of friction coefficient/tangential force versus the imposed amplitude as a function of the number of cycles were recorded as fretting logs, as shown in Fig. 2(a–i). Fretting log diagrams were used to determine the contact interface states during the fretting test. When the numbers of the cycles are 1000, 5000 and 9900, the fretting log diagrams all took on a quasi-rectangular shape, demonstrating that the fretting followed a gross slip regime during the whole test both in air and in Hank’s solution. This indicated that wear occurred at the interface of the JDBM and the contact ball, and the two contact surfaces were relatively moving around all the contact area. The area within the fretting loop is equal to energy (E_d_) dissipated to a contact surface (Friction coefficient should be multiplied by normal load). Figure 2(j) shows the evolution of dissipated energy with cycles in all testing environments. It was observed that the evolution of dissipated energy contained two distinct stages. In the first stage, dissipated energy was comparatively higher than that in the secondary stage. Dissipated energy remained almost steady in the secondary stage. Besides, dissipated energy increased with the increase of normal load both in air and in Hank’s solution. By contrast, the dissipated energy in Hank’s solution containing 10% FBS were a little bit lower than that in Hank’s solution and much lower than that in air.

### Surface damage analysis

**Figure 3:**
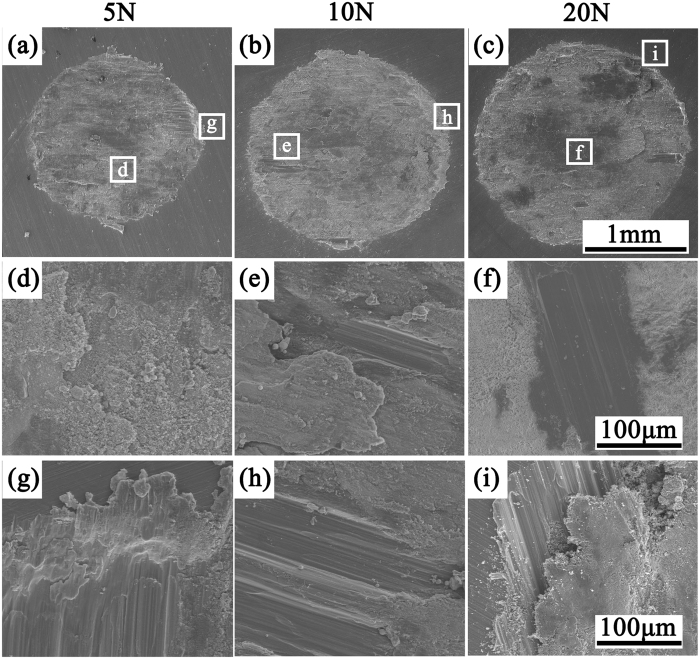
SEM morphologies of wear scars of JDBM with the normal load of 5 N, 10 N and 20 N in air and enlarged images of (**d**–**f**) the center region; (**g**–**i**) the edge region.

**Figure 4:**
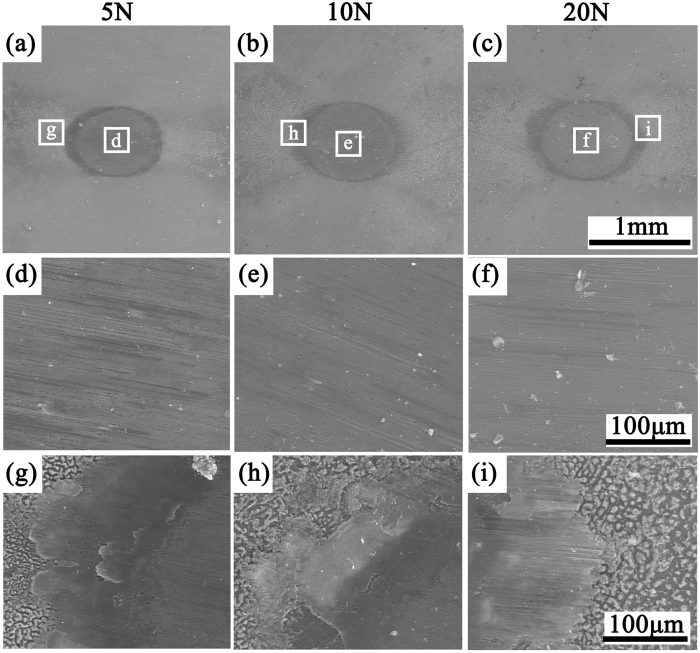
SEM morphologies of wear scars of JDBM with the normal load of 5 N, 10 N and 20 N in Hank’s solution without addition of FBS and enlarged images of (**d**–**f**) the center region; (**g**–**i**) the edge region.

**Figure 5:**
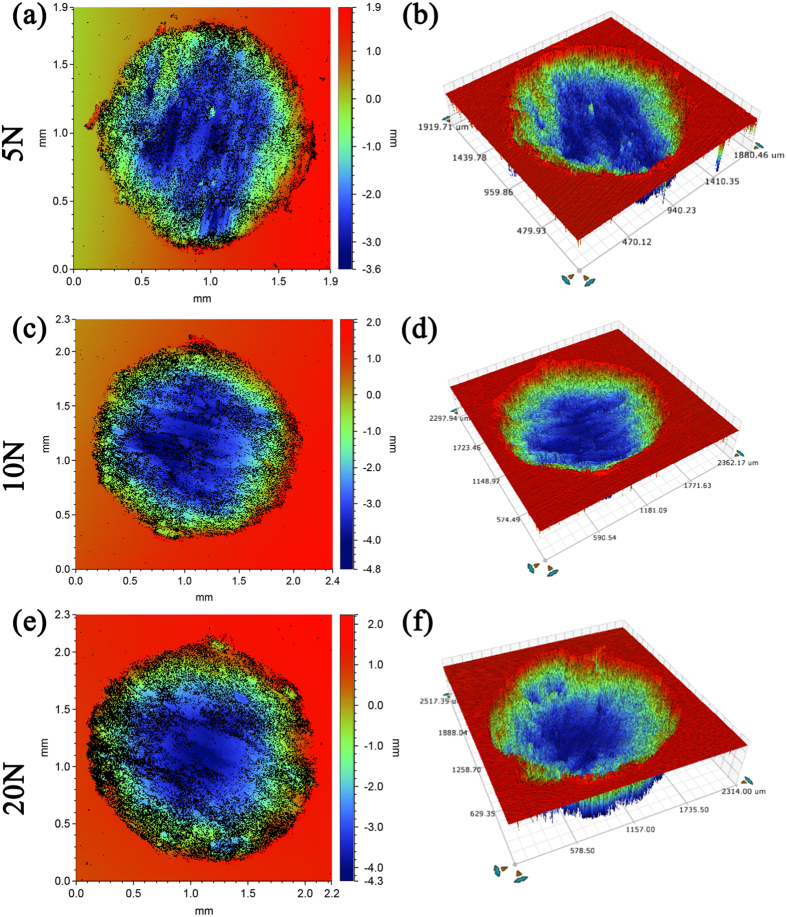
2-D (**a**,**c**,**e**) and 3-D (**b**,**d**,**f**) fretting scar with different colors representing different depth in air.

Figure 3 showed the representative SEM micrographs of fretted surfaces of the flat specimens after fretting in air. The SEM micrographs of fretted surface in Hank’s solution without FBS were shown in Fig. 4 and those in Hank’s solution with FBS were shown in Fig. S1. It can be found that the area of the wear scar significantly improved with the increase of normal load in air, while it only slightly increased in Hank’s solution (Figs 3(a–c), 4(a–c) and S1(a–c)). The details of wear scars at center were similar in air under 5 N, 10 N and 20 N normal load (Fig. 3(d–i)). Both scratch and delamination were observed when fretting occurred in air (Fig. 3(d–i)). The direction of scratch marks was all parallel to the sliding direction. Materials peeled off from the surface were in sheet shapes. Some debris moved out of the wear scar, while others still stayed in the hole and even remained attached to the surface. Although in Hank’s solution the details of wear scars under 5 N, 10 N and 20 N were very close to each other, there were no delamination but only shallow and parallel scratches. When FBS added to Hank’s solution, the detail of wear scar is similar to that in Hank’s solution without FBS (Fig. 4 and S1). Wear scars and debris generated in air were black to the unaided eyes, while wear scars in Hank’s solution were gray. In addition, there was no evident debris during the fretting in Hank’s solution. The debris accumulating at the edge of the scar, and could not be easily moved because of the corrosion behavior (Fig. 5 and S1). The wear scars in Hank’s solution were much smoother than that in air. All of these results were consistent with the evolution of friction coefficient in Fig. 1, which will be discussed in details in the discussion section.

### Damage analysis of three dimension

**Figure 6:**
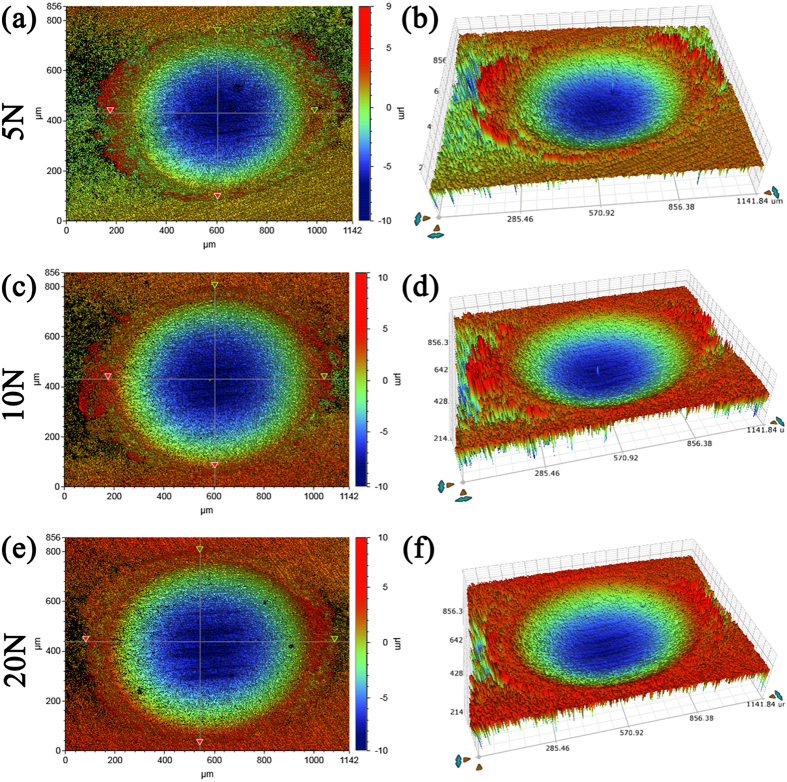
2-D (**a**,**c**,**e**) and 3-D (**b**,**d**,**f**) fretting scar with different colors representing different depth in Hank’s solution without addition of FBS.

Figure 5 shows 2-D and 3-D fretting scar in air with different color indicating different depth. Figure 6 shows these in Hank’s solution and Fig. S2 shows these in Hank’s solution with addition of FBS. It is observed that the area of the wear scar increased with the increase of normal load in air. Besides, the holes derived from fretting in air were much deeper than those in Hank’s solution. Moreover, the bottom of the holes in Hank’s solution was smoother than that in air. No significant difference was observed between the 2-D and 3-D morphology of wear scars in simulated fluid with and without FBS except the size of the wear scar.

**Figure 7:**
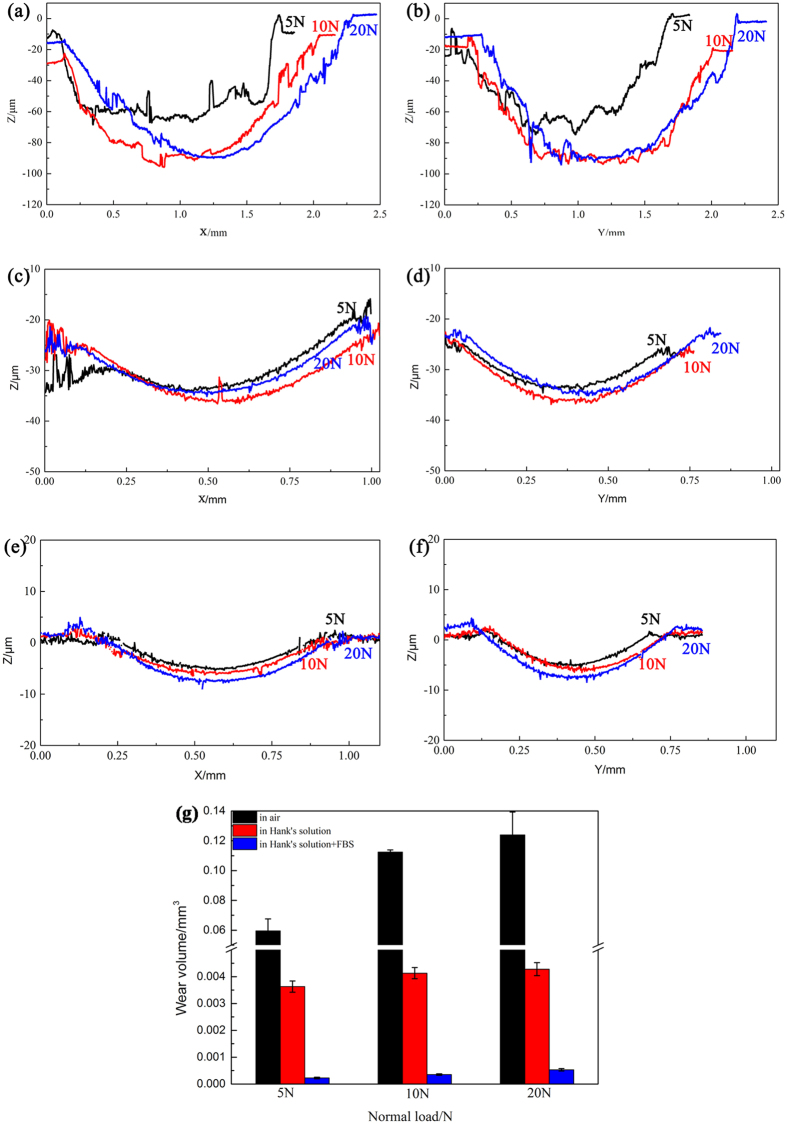
Depth profile parallel and perpendicular to the sliding direction at the maximum width of the wear scars of JDBM with the normal load of 5 N, 10 N and 20 N in air and in Hank’s solution, respectively. (**a**,**b**) in air; (**c**,**d**) in Hank’s solution without addition of FBS; (**e**,**f**) in Hank’s solution with addition of FBS. (**g**) The wear out volume of JDBM with the normal load of 5 N, 10 N and 20 N in air (black), in Hank’s solution without addition of FBS (red), and in Hank’s solution with addition of FBS (blue), respectively.

The depth profiles of the wear scar in air and in Hank’s solution were shown in Fig. 7. As the normal load increased, the maximum depth of the wear scar increased significantly in air and slightly in Hank’s solution. The maximum depth of the wear scar in air was around 60 μm under 5 N, 80 μm under 10 N and 100 μm under 20 N. Meanwhile, the maximum depth of the wear scar was around 10 μm in Hank’s solution. After addition of FBS, the maximum depth of the wear scar was less than 10 μm. It can be clearly showed that a smoother wear surface in Hank’s solution than that in air.

Furthermore, the wear volume increased with the normal load obviously in air and slightly in the Hank’s solution (Fig. 7(g)). The wear volume in Hank’s solution was about one order of magnitude lower than that in air (Fig. 7(g)). The wear volume in Hank’s solution with addition of SBF was about one order of magnitude lower than that in Hank’s solution without the addition of FBS. Hank’s solution significantly reduced the wear damage of JDBM, and FBS also reduced the wear damage.

### Surface composition analysis

**Figure 8:**
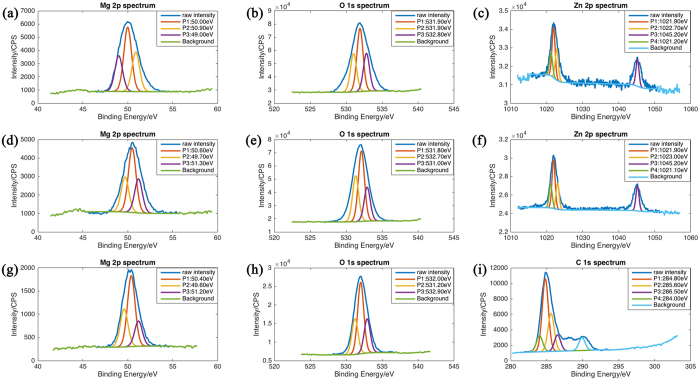
XPS spectra of Mg 2p, O 1s, Zn 2p and C 1s from the wear tracks on JDBM specimens after fretting (**a**–**c**) in air, in Hank’s solution with FBS (**d**–**f**) and without FBS (**g**–**i**).

Besides its physical influences, Hank’s solution and SBF had chemical effects on the contact surface. The EDS spectrum in the fretting area in air and in Hank’s solution is shown in Fig. S3–S4. It shows O content in the fretted zone was much higher than in other parts of the surface, indicating that a MgO layer formed during fretting. Hank’s solution displayed slightly higher O content in the center of fretted zone than in the unfretted zone (Fig. S4). The O content of debris-accumulated zone (51.30 wt%, Fig. S4(a)) was higher than that of the wear scar fretted in air (43.82 wt%, Fig. S4(b)), demonstrating that the solution promoted the change of surface chemical composition. In addition, Ca, P and C were also detected at the edge of the wear scar, indicating chemical reaction at the interface. However, the Ca and P contents were very low. The occurrence of C may result from the reaction between Mg(OH)_2_ or MgO and CO_2_ from air after the tests. A more precise characterization of the fretting surface was evaluated by XPS. XPS spectra of Mg 2p, O 1s, Zn 2p and C 1s from the wear tracks on JDBM specimens after fretting was shown in Fig. 8. In Fig. 8(a), the position of the Mg 2p peak (50.90 eV) corresponds to MgO^39^. The other two peak of Mg correspond to Mg^40^. Oxygen peaks in Fig. 8(b) situated at an approximate energy of 531 eV are characteristic of metal oxides^41^. The position of the Zn 2p peak (1021.10 eV) corresponds to ZnO^40^. These results indicated that during the fretting tests in air, Mg and Zn reacted with oxygen. When fretting occurred in Hank’s solution, also MgO and ZnO existed on the surface of JDBM alloy according to the XPS spectra (Fig. 8(d–f)). However, the content of these metal oxides was higher in Hank’s solution than that in air. This indicated that Hank’s solution promoted the corrosion of JDBM alloy. The C 1s peaks in Fig. 8(i) at around 285.0 eV originating from C–(C, H) bonds^40^ were detected in presence of FBS. This indicates adsorption of protein on the metal surface. The O 1s at approximately 532.0 eV correspond to organic compounds^40^. The adsorbed layer of protein contributed to the reduction of the fretting wear.

## Discussion

Fretting resistance is not an intrinsic property of a material, or even of a material couple. For example, depending on the amplitude of the displacement or on the normal load, the fretting condition can be partial slip, gross slip or mixed slip. These three situations induce different local loading on the surface. Moreover, the environmental condition may have a large effect^42^.

The friction coefficients basically decreased with the increase of normal load both in air and in Hank’s solution in our study. However, the evolutions of friction coefficients with number of cycles were similar in air under 5 N, 10 N and 20 N normal load or in Hank’s solution (Fig. 1). This means that the normal loads in these tests led to similar evolution of sliding conditions. The area of the wear scar increased with the increase of normal load significantly in air and slightly in Hank’s solution. As the normal load increased, the maximum depth of the wear scar increased also significantly in air and slightly in Hank’s solution (Fig. 7). Besides, both the dissipated energy and the wear volume increased with the normal load obviously in air and slightly in Hank’s solution (Fig. 7(g)). The effect of normal load on the fretting damage in air should be taken into serious consideration. This results are consistent with the conclusion that normal load is one of the key factors on the fretting behavior^18^. By contrast, Hank’s solution has lubrication ability that alleviates the effect of increase of normal load.

In fact, the normal load and the amplitude of the displacement can lead to three different fretting regimes: partial slip regime (partial slip condition is maintained throughout the test duration), gross slip regime (gross slip condition is maintained throughout the test) and mixed slip regime (sliding condition changes)^42^. However, in present tests, the normal loads, displacement amplitude and environment condition all caused gross slip between the two contact surfaces.

Mechanically assisted corrosion is a continuing concern of all metallic implant materials^43^. The conjoint degradation processes of corrosion and wear of metal surfaces is clearly of great importance in the design of implants. It is also clear that in a situation where corrosion and fretting are both possible degradation mechanisms, each could have a profound effect on the other.

**Figure 9:**
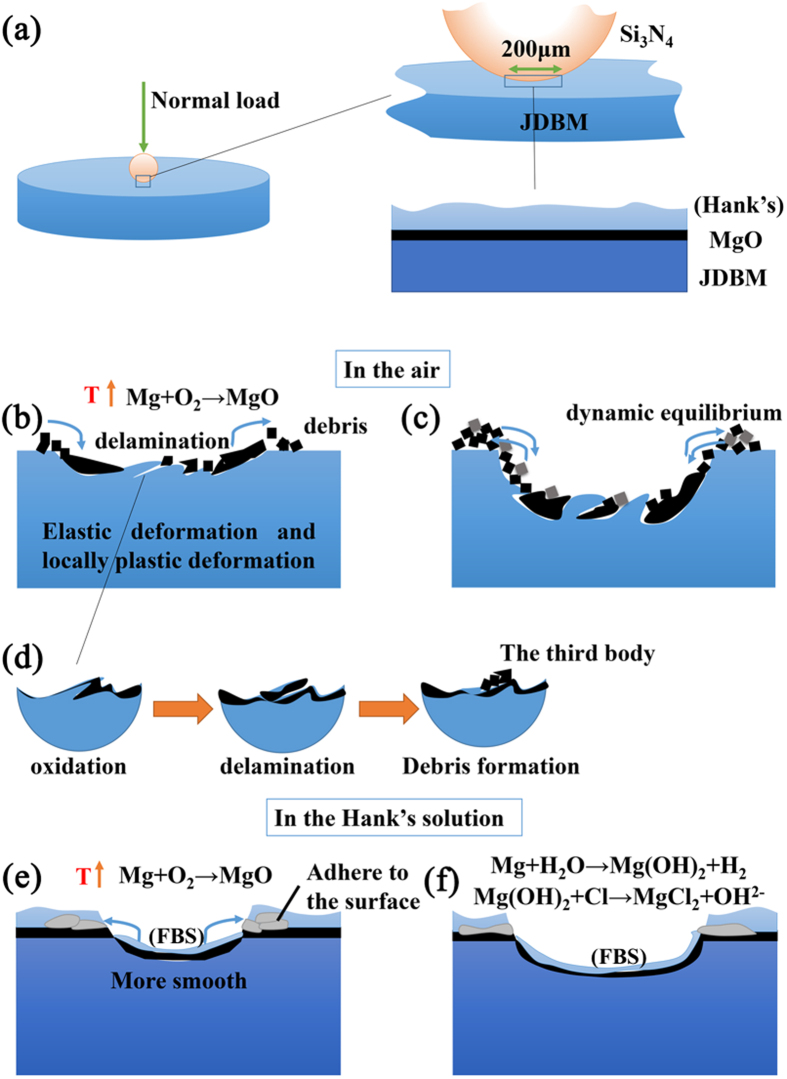
Illustration of the damage mechanism of JDBM in air and in Hank’s solution. (**a**) The contact state between the two surface before fretting; (**b**,**c**) the physical and chemical change during the fretting in air; (**d**) the formation of “the third body” in air; (**e**,**f**) the physical and chemical change during the fretting in Hank’s solution.

The results of this study showed that the fretting behaviors of JDBM in a simulated physiological environment were quite different from those in air. Figure 9 illustrated the damage mechanism of JDBM in air and in Hank’s solution. In air, the evolution of friction coefficient can be divided into two stages. In the first stage the friction coefficient was increased quickly due to the destruction of a natural oxidation layer on the alloy surface, which was also showed in ref. 44. As the protective layer was gradually removed, the friction coefficient increased because adhesion wear and plastic deformation occurred in local area^44^. The initial surface is not perfectly smooth, so the real contact pressure was higher than the initial maximum Hertzian contact pressure. The higher pressure is one of the reasons that can cause adhesion wear and plastic deformation. The real contact area enlarged with the cycle number. Local material surface became brittle and could be easily destroyed to form debris (Fig. 9(d)). This process is called “delamination”^45^,^46^. Plastic deformation and oxidation are both responsible for the embrittlement of the material surface. Then the friction coefficient descended since wear debris detached from the materials and prevented adhesion wear, transforming the “two bodies contact (the contact of initial surface of the two material)” into “three bodies contact (debris were between the initial two surface)”^47^. Since the local temperature near the fretted zone was elevated due to the mechanical friction, the surface was oxidized and debris was mainly composed of magnesium oxide (Fig. 8(b)). The oxide debris was continuously made and removed from the surface and got crushed between the surfaces during the repeating mechanical action. In the second stage the friction coefficient fluctuated around a constant value when the formation and removal of debris maintained equilibrium (Fig. 8(c)). However, the depth and area of the wear scar changed in the process of the fretting, and the initial contact load also decreased gradually, leading to the variation of friction coefficient.

When the fretting happened in an aqueous solution, the solution played triple roles on JDBM. Firstly, the solution acted as a lubricant that prevented the two fretting surfaces from direct contact and thus reduced the fretting damage (Fig. 8(f)). Secondly, it prevented large increase of temperature of the fretted zone and thus protected the fretted surface from severe oxidation. Thirdly, the solution was a corrosion media that accelerated the deterioration of the material. Therefore, the solution had contrary effects on the fretting behavior of JDBM. Based on results of the present study, compared to fretting in air, Hank’s solution played more of a protector role as it reduced friction coefficients of JDBM by a factor of 5 and the wear out volume by one order of magnitude. From Fig. 1 it can be seen that the evolution of friction coefficients in Hank’s solution only had one stage. The friction coefficient had a stable value because the contact surface was much smoother and there was no debris in the center of the wear scar. After the destruction of oxidation layer, the surface material was delaminated in a smaller scale compared to that happened in air. The debris was removed from the contact surface and firmly attached to the edge of the wear scar. At the same time, the magnesium alloy was corroded in the solution. The friction coefficient increased slightly since both fretting and corrosion developed.

SBF may play two roles in fretting behavior. One is that SBF has an influence on the corrosion behaviors of the alloy. Previous studies showed that albumin certainly affects the corrosion behavior of Mg and its alloys, but this influence can be drastically alloy-dependent^48^. Previous studies also showed that the role of proteins, especially albumin was an essential factor affecting corrosion and fretting corrosion of metal^49^. It has been reported that albumin acted as an inhibitor against corrosion degradation during the fretting^50^. Another is that 10% bovine serum can improve the lubrication action of Hank’s solution. XPS spectra indicated that protein attached to the material surface. The friction coefficients, dissipated energy and wear volume of fretting test in Hank’s solution with 10% FBS were less than those recorded during the fretting test in Hank’s solution without FBS. FBS was as a lubricant decreasing the wear damage.

Our study also calculated the dissipated energy of fretting. After the initial stage where higher energy dissipation compared to that in second stage was observed, dissipated energy was linearly accumulated with respect to number of cycles, because dissipated energy remained constant during fretting cycles. The evolution of dissipated energy was corresponding to that of friction coefficient, indicating that the initial stage, destruction of the metal surface, stayed in a very short period. The wear volume increased with the increase of the dissipated energy under various normal loads both in air and in Hank’s solution.

The debris is important when fretting happens in air. They are called “the third body”, governing the fretting wear in some extent^51^. Wear debris may influence the fretting behavior due to accumulation at the center of the wear scar. Metal oxide adhered to the surface of the spherical specimen to the unaided eye. The adhesion had a big influence on the fretting wear by changing the contact states of the two surfaces. The surface became rough for both JDBM and Si_3_N_4_ ball. Debris can also move out or move in the scar, so the coefficient of friction did not remain a constant but fluctuated slightly after several thousands cycles. In physiological environment debris could cause adverse biological reactions, such as immunobiologic reaction^52^. Macrophage accumulated to the surface swallow the debris by phagocytosis. This can stimulate the formation of osteoclasts resulting in osteolysis. But the results of fretting behavior of JDBM alloy in Hank’s solution showed that there are no dispersed debris around the wear scar possibly due to the corrosion effect of Hank’s solution and biodegradable behavior of JDBM alloy. It suggested that biodegradable JDBM alloy may not produce dispersed debris when fretting occurs in body fluid. Maybe this is another advantage of biodegradable magnesium over traditional non-degradable metallic biomaterials when they are used as bone implant, but there are still many other factors to consider in the physiological environment.

Although fretting damage of JDBM alloy in aqueous solution is much less than that in dry condition, it causes big concern. With their lower hardness compared to traditional metallic biomaterials such as stainless steel, titanium and CoCrMo alloys, magnesium alloys exhibit poorer fretting resistance. For example, maximum wear scar depth of a Ti-6Al-4V alloy after self-mating wear for 9480 circles under a load of 15 N in dry condition is 15.8 ± 1.7 μm^53^, which is much less than JDBM alloy in air and similar to JDBM in Hank’s solution in this study. Another research pointed out that the coefficient of friction of most Ti-based alloys under 10 N normal load for 10000 cycles with relative displacement of 80 μm and the frequency at 10 Hz in Hank’s solution was in the range of 0.46–0.50^14^, which is a little higher than the JDBM alloy with the relative displacement of 200 μm. The Hank’s solution has a more effective lubrication for JDBM to Ti-based alloys. These damage behaviors were not easy to be compared because of the difference of relative displacement.

Abrasion and cracking are the predominant wear mechanisms for Ti-based alloys during fretting in Hank’s solution^14^. Because of the biodegradability of magnesium alloys, in other words, the corrosion behavior in Hank’s solution, oxidation is another main wear mechanism for JDBM alloy. Competition between local contact fatigue and wears decides whether crack can be caused^54^. In this research, the velocity of delamination is higher than that of cracking, so there is no crack under the wear scar when cross-section morphologies were observed through using SEM.

It was reported that the mechanical properties of present orthopedic implants should last for 1–3 months. When utilizing the poor corrosion resistance of magnesium alloys as biodegradable implant materials, it is necessary to take fretting as well as corrosion rate and their interrelationship into consideration to estimate their valid life. Fretting corrosion is of increasing concern in orthopaedic surgery and needed to be further researched.

The physiological environment is really complex because it includes many other chemical or biological substance and the real fretting mode is not only tangential fretting, but also could be radial fretting, rotational fretting, torsional fretting or even mixture of these modes. More detailed research should be done to investigate the fretting behaviors of JDBM alloy. Our present study of the fretting behaviors of biodegradable magnesium in Hank’s solution with and without FBS showed the important effect of Hank’s solution and SBF. It can be a stepping stone to further study.

## Conclusions

The fretting behaviors of JDBM alloy in the air and in Hank’s solution with and without FBS under 5 N, 10 N and 20 N normal loads with a displacement of 200 μm under a frequency of 10 Hz at 37 °C in air and in Hank’s solution were investigated. The possible mechanisms of fretting damage were mentioned. We have the following conclusions.

Hank’s solution reduced friction coefficients of JDBM by a factor of about 4 and altered the evolution trend of friction coefficients with time. The wear volume of JDBM in Hank’s solution was about one order of magnitude lower than that in air. Hank’s solution could reduce the damage of JDBM significantly during fretting. Furthermore, the presence of FBS could reduce the fretting damage by adsorption of protein on the surface of JDBM alloy.

The friction coefficient decreased with the increase of normal load and the wear out area increased with the increase of normal load significantly in air and slightly in Hank’s solution. Normal load is one of the key factors during fretting, however, the influence of that is evident in air. The wear volume increased with the increase of dissipated energy. Dissipated energy bridges the gap between the movement and the wear damage.

When JDBM fretted in air, abrasive wear, delamination and oxidation were predominant wear mechanisms. Delamination and oxidation wear were predominant wear mechanism in Hank’s solution. Debris did not involve in the fretting in Hank’s solution.

Although Hank’s solution reduced the fretting behavior of JDBM alloy, fretting accelerated their corrosion behavior slightly. FBS could protect the surface by forming a protein layer.

## Materials and Methods

### Materials and specimen preparation

JDBM alloy was prepared by semi-continuous casting with high purity Mg (≥99.99%), Zn (≥99.995%), Mg-25%Nd and Mg-30%Zr, which was detailed in our previous report^55^. The as-received materials were machined into cylindrical specimens of 24 mm in diameter and 7.9 mm in thickness in order to fit size requirement of the test system. The flat surfaces of cylindrical specimens were mechanically polished up to 5000 grit to 0.23 μm, followed by ultrasonic cleaning in acetone, and absolute ethanol for 10 min each. The spherical specimens of Si_3_N_4_ (roughness is 0.014 μm) with a diameter of 10 mm were used to form the ball-on-flat configuration. The hardness of Si_3_N_4_ is very high and one order of magnitude higher than that of JDBM, assuring nearly no damage occurring on the Si_3_N_4_ sphere and making it easy to focus on fretting behavior of JDBM alloy.

### Fretting tests

Self-mating *in vitro* fretting tests in air and in Hank’s solution with and without FBS were conducted in a ball-on-flat configuration using an SRV^®^4 test system (Optimol, Germany). Hank’s solution consisted of NaCl 8.00 g, KCl 0.40 g, CaCl_2_ 0.14 g, NaHCO_3_ 0.35 g, MgSO_4_·7H_2_O 0.20 g, Na_2_HPO_4_·12H_2_O 0.12 g and KH_2_PO_4_ 0.06 g per litre. The applied normal load were 5 N, 10 N and 20 N, with corresponding initial maximum Hertzian contact pressure of 106 MPa, 133 MPa and 168 MPa. The tests were carried out with a displacement of 200 μm under the frequency of 10 Hz for a period of 1000 s at 37 °C. Each test was repeated three times (n = 3). The friction coefficients were kept recording during the tests while the fretting logs were recorded for 1 second every 30 seconds.

### Damage analysis

After fretting tests, the materials were cleaned by ultrasonic cleaning in acetone, and absolute ethanol for 10 min each. The worn scars on cylindrical specimens were observed by means of environmental scanning electron microscope (ESEM, Quanta 200FEG) and surface 3D profiler based on white light interferometry (NPFLEX, Germany).

## Additional Information

**How to cite this article**: Li, W. *et al*. Fretting properties of biodegradable Mg-Nd-Zn-Zr alloy in air and in Hank’s solution. *Sci. Rep.* **6**, 35803; doi: 10.1038/srep35803 (2016).

**Publisher’s note:** Springer Nature remains neutral with regard to jurisdictional claims in published maps and institutional affiliations.

## Supplementary Material

Supplementary Information
