# Supplementary material for: Fretting properties of biodegradable Mg-Nd-Zn-Zr alloy in air and in Hank’s solution
Source: Sci Rep. 2016 Nov 4;6:35803. doi: 10.1038/srep35803 (PMC5095712; doi:10.1038/srep35803)

**Fretting properties of biodegradable Mg-Nd-Zn-Zr alloy in air and in Hank’s solution**

Wenting Li^a,1^, Nan Li^a^, Yufeng Zheng^a^,* GuangYin Yuan^b^**

^a^ Department of Materials Science and Engineering, College of Engineering, Peking University, Beijing 100871, China

^b^ National Engineering Research Center of Light Alloys Net Forming, Shanghai Jiao Tong University, Shanghai 200240, China


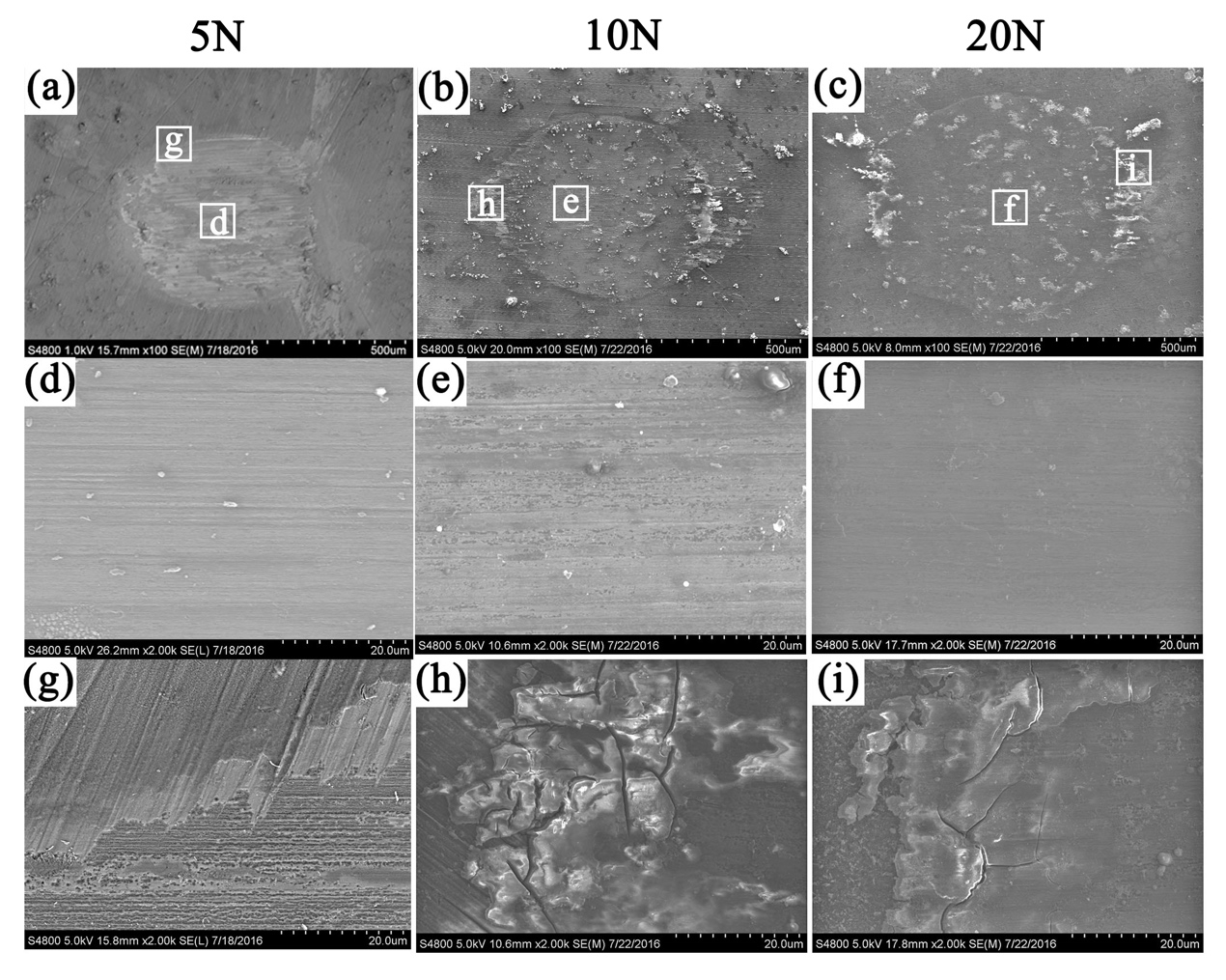


Supplementary Figure.S1 SEM morphologies of wear scars of JDBM with the normal load of 5 N, 10 N and 20 N in Hank’s solution with addition of FBS and enlarged images of (d)(e)(f) the center region; (g)(h)(i) the edge region.


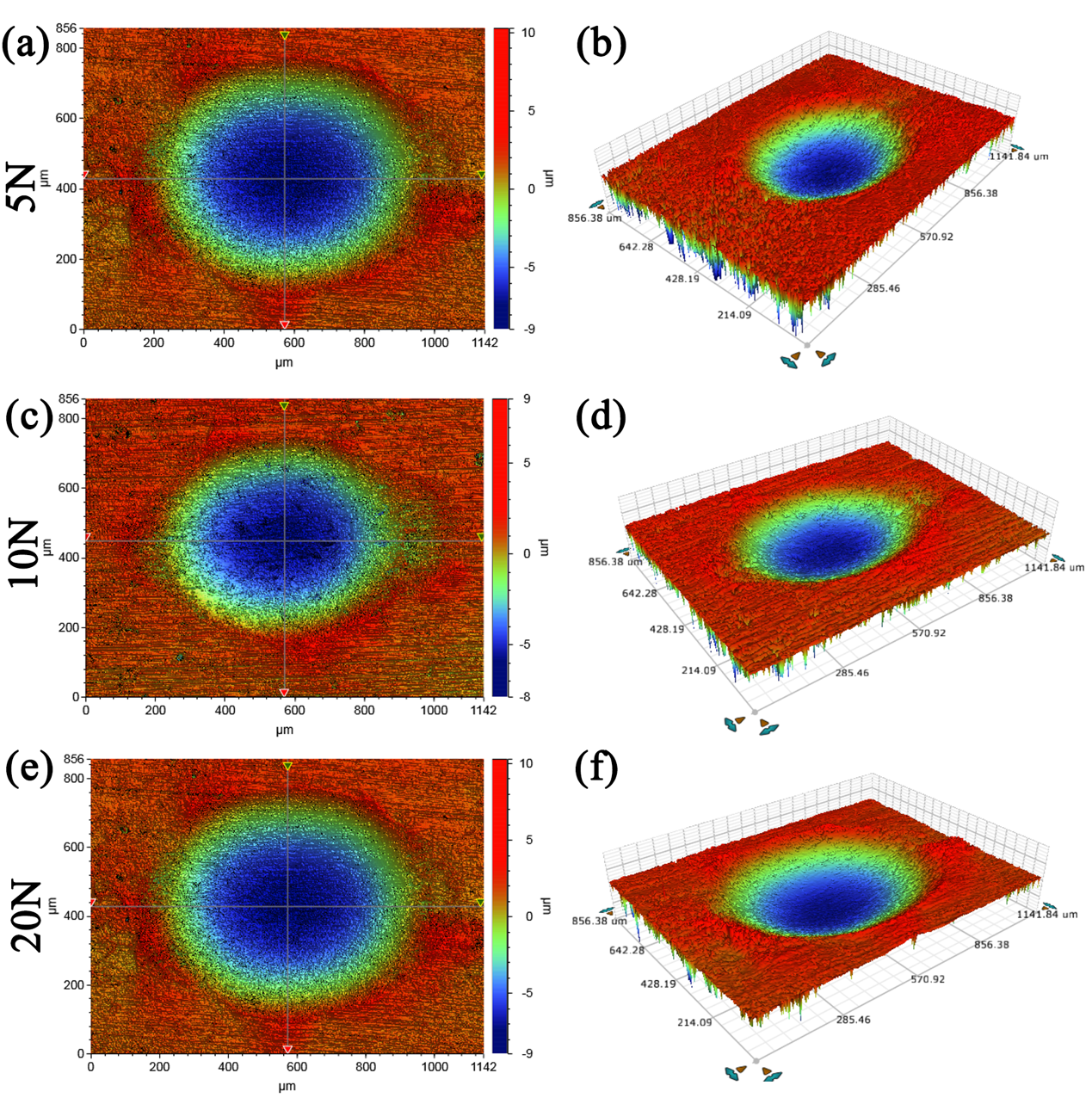


Supplementary Figure.S2 2-D (a)(c)(e) and 3-D (b)(d)(f) fretting scar with different colors representing different depth in Hank’s solution without addition of FBS.


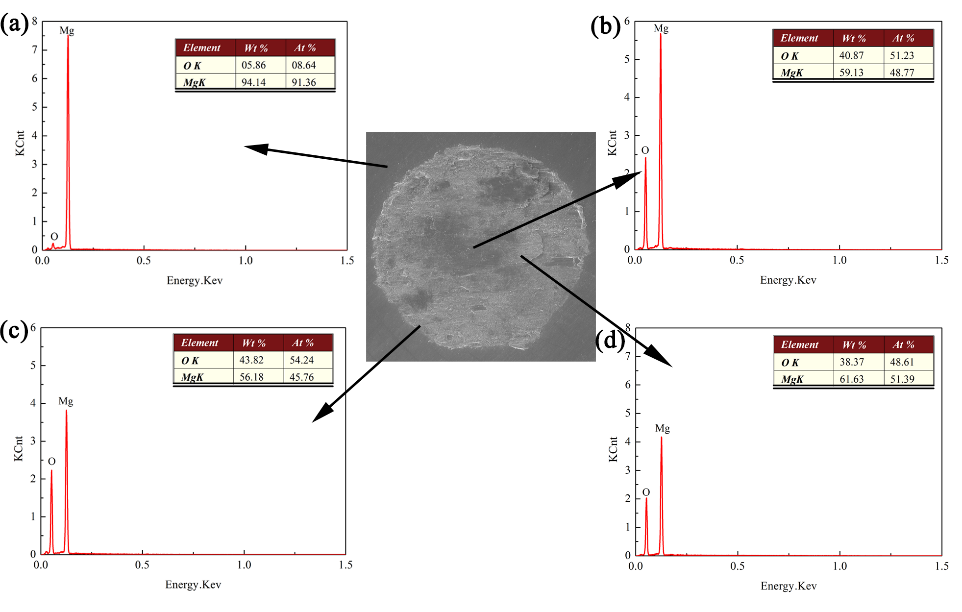


Supplementary Figure.S3 EDS spectrum of the fretting scar in air. (a) far from the wear scar; (b) the center of the wear scar (black); (c) the edge of the wear scar; (d) the center of the wear scar (white).

Supplementary Figure.S4 EDS spectrum of the fretting scar in Hank’s solution. (a) the edge of the wear scar; (b) the center of the wear scar.


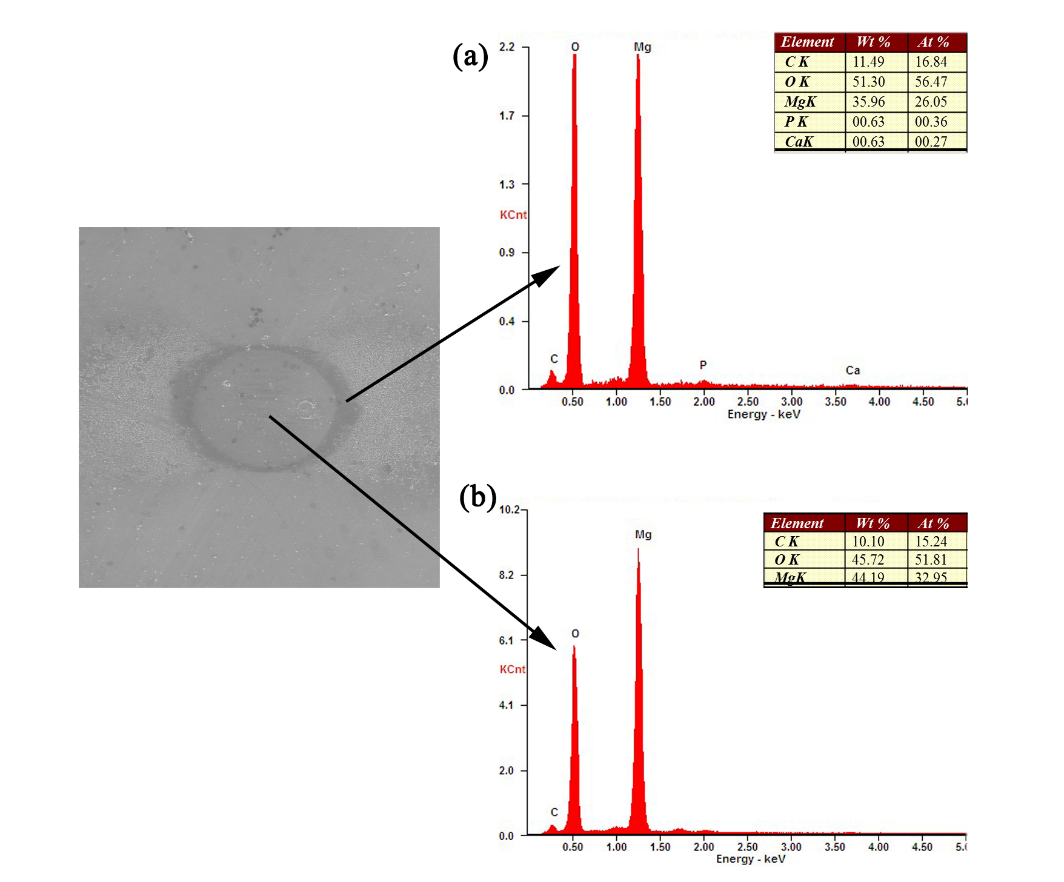

Supplement: Supplementary Information [file srep35803-s1.doc]
